# Wave energy devices with compressible volumes

**DOI:** 10.1098/rspa.2014.0559

**Published:** 2014-12-08

**Authors:** Adi Kurniawan, Deborah Greaves, John Chaplin

**Affiliations:** 1 School of Marine Science and Engineering, Plymouth University, Plymouth PL4 8AA, UK; 2 Faculty of Engineering and the Environment, University of Southampton, Southampton SO17 1BJ, UK

**Keywords:** wave energy, compressible volume, axisymmetric device, numerical modelling, absorbed power

## Abstract

We present an analysis of wave energy devices with air-filled compressible submerged volumes, where variability of volume is achieved by means of a horizontal surface free to move up and down relative to the body. An analysis of bodies without power take-off (PTO) systems is first presented to demonstrate the positive effects a compressible volume could have on the body response. Subsequently, two compressible device variations are analysed. In the first variation, the compressible volume is connected to a fixed volume via an air turbine for PTO. In the second variation, a water column separates the compressible volume from another volume, which is fitted with an air turbine open to the atmosphere. Both floating and bottom-fixed, axisymmetric, configurations are considered, and linear analysis is employed throughout. Advantages and disadvantages of each device are examined in detail. Some configurations with displaced volumes less than 2000 m^3^ and with constant turbine coefficients are shown to be capable of achieving 80% of the theoretical maximum absorbed power over a wave period range of about 4 s.

## 1. Introduction

It is well known that a heaving axisymmetric wave energy device in the open sea is potentially capable of absorbing all the wave power that is incident on a wavefront λ/2*π* wide, where λ is the wavelength [1–3]. For regular 8-s, 1-m amplitude, waves, this amounts to a potential of about 500 kW of power absorbed by a single device. The condition is that the device resonates with the waves, with the optimum amplitude [4,5].

The resonance period of a heaving body, however, is governed by its waterplane area and its mass. To resonate at 8 s, for example, a heaving semi-submerged sphere would need a diameter of approximately 30 m, equivalent to a displaced volume of 7000 m^3^. On the other hand, although, to resonate at 8 s, a heaving circular cylinder can have less volume than the sphere, the resonance bandwidth would be narrower. Unless some means of phase control is used, a heaving body has to be sufficiently large to resonate at frequencies typical of ocean waves as well as to have a satisfactorily broad bandwidth.

The above is true if the heaving body is rigid. However, if we allow the submerged volume of the heaving body to be compressible, then the rate of change of its buoyancy with heave, i.e. its hydrostatic stiffness, is lowered. As a lower stiffness means a longer resonance period for the same mass, with a compressible volume it is possible to achieve resonance with a smaller device. Moreover, for two systems with equal resonance periods, the bandwidth of the system with a lower stiffness would be broader than that of the one with a higher mass, provided the two systems have the same damping.

Motivated by this idea, Farley [6,7] recently proposed an air-filled compressible wave energy device in the form of a heaving wedge which opens and closes as it heaves. As it does so, air is pumped into and out of a fixed volume which is connected to the compressible volume via a self-rectifying turbine. The resonance period of the device is tunable by adjusting the stiffness of the wedge, which is governed by the air volume inside. An axisymmetric version of the device has also been proposed [8]. Several other devices used some kind of flexible volume, although not necessarily motivated by the same idea. A device resembling one of the devices which we shall analyse below was proposed as early as 1974, although a mathematical analysis was not presented [9], §2.3. The Lancaster flexible bag was developed at around the same time [10,11], as also the SEA Clam [12]. Both were of the order of a wavelength long, and both used flexible bags to pump air through a turbine. More recently, the Archimedes Wave Swing, a comparatively smaller device in the form of a completely submerged cylinder with a movable top, was proposed and developed [13]. Instead of an air turbine, the device used a linear generator to convert the absorbed wave power directly from vertical oscillations of the movable top. Another flexible device which has been under development recently is the Anaconda, a long rubber tube filled with water [14,15]. It captures wave energy by bulge wave, which is associated with longitudinal oscillations of water inside the tube.

This paper concerns air-filled compressible wave energy devices which absorb wave energy via vertical motions. All devices considered here are axisymmetric, with horizontal extents much smaller than the operating wavelengths. We first look at the more fundamental problem of bodies with compressible volumes but without power take-off (PTO) systems, to study the effects a compressible volume can have on the body response. The volume is filled with air, and variability of volume is achieved by means of a horizontal surface free to move up and down relative to the body. We consider both fixed and floating bodies and study the effects of having the wetted side of the moving surface facing up or down.

By including suitable PTO systems, we can transform these compressible bodies into wave energy devices, and these are considered next. Two device variations are investigated. In the first variation, the compressible volume is connected to a fixed volume via a self-rectifying air turbine. In the second variation, a water column separates the compressible volume from another volume, which is fitted with an air turbine open to the atmosphere. The water column replaces the fixed volume in providing the required restoring force on the compressible volume. Both bottom-fixed and floating device configurations are considered. The former is more suited for near shore locations, while the latter can operate further offshore. Linear, frequency-domain, analysis is employed throughout, where the hydrodynamic parameters are obtained from linear potential theory and no losses are taken into account. Numerical results in the form of device displacements and absorption widths for some representative device dimensions are presented. Based on these, the advantages and disadvantages of each device are discussed in detail.

## 2. Preliminaries

Before deriving the equations specific to each compressible body or device, we shall summarize some of the more general equations applicable to all bodies and devices considered in this paper.

We employ a Cartesian coordinate system, where the mean free surface is *z*=0 and the incident wave propagates in the positive *x*-direction. Time-harmonic motions of small amplitudes are considered, with the complex factor *e*^i*ωt*^ applied to all oscillatory quantities, where *ω* is the angular frequency.

As the *x*-axis has been chosen to be parallel to the incident wave direction, only three modes are necessary to describe the rigid body motions, namely surge, heave and pitch (*j*=1,3,5). In addition to the conventional rigid body modes, we have one additional mode (*j*=7) defined as the vertical displacement of the moving surface *S*_*s*_ relative to the body. In the following, we shall restrict our attention to vertical motions only (*j*=3,7). Within linear theory, only the vertical motions contribute to power absorption.

Following the generalized modes method of Newman [16], we define each mode by a vector ‘shape function’ **S**_*j*_(**x**) with Cartesian components *u*_*j*_(**x**),*v*_*j*_(**x**) and *w*_*j*_(**x**). For heave (*j*=3), the shape function **S**_3_ is simply a unit vector in the vertical direction. For the additional mode (*j*=7), the shape function **S**_7_ is given as

2.1 $$u_{7}=0,\;v_{7}=0\;\mathrm{and}\;w_{7}=\left\{ \begin{array}{ll} 1 & \text{for }\text{x}\in S_{s}\\ 0 & \text{elsewhere}. \end{array}\right.$$

The displacement of an arbitrary point **x** within the body is given by $\sum_{j}\xi_{j}{\text{S}}_{j}(\text{x})$, where *ξ*_*j*_ is the complex displacement amplitude of the body in mode *j*. The normal component of **S**_*j*_(**x**) on the wetted body surface *S*_b_ is expressed as

2.2 $$n_{j}(\text{x})={\text{S}}_{j}(\text{x})\cdot \text{n}(\text{x})=u_{j}(\text{x})n_{x}(\text{x})+v_{j}(\text{x})n_{y}(\text{x})+w_{j}(\text{x})n_{z}(\text{x}),$$

where the unit normal vector **n** points out of the fluid domain and into the body. The normal component of **S**_7_ on the wetted body surface is therefore

2.3 $$n_{7}={\text{S}}_{7}\cdot \text{n}=\left\{ \begin{array}{ll} n_{z} & \text{for }\text{x}\in S_{s}\\ 0 & \text{elsewhere}. \end{array}\right.$$

In accordance with linear theory, the generalized pressure force corresponding to mode *j* is defined as

2.4 $$F_{j}=\iint_{S_{b}}pn_{j}\;dS=-\rho \iint_{S_{b}}(i\omega \phi +gz)n_{j}\;dS,$$

where the first term is the hydrodynamic contribution and the second term is the hydrostatic contribution. Here, *ϕ* is the total velocity potential, which may be expressed as the sum of the diffraction potential *ϕ*_*D*_ and the radiation potential *ϕ*_*R*_. The diffraction potential *ϕ*_*D*_ is defined as the sum of the incident wave potential *ϕ*_*I*_ and the scattering potential *ϕ*_*S*_.

The contribution from the diffraction potential is defined as the wave excitation force *F*_*ej*_

2.5 $$F_{ej}=-i\omega \rho \iint_{S_{b}}\phi_{D}n_{j}\;dS=-i\omega \rho \iint_{S_{b}}\phi_{D}\frac{\partial \phi_{j}}{\partial n}\;dS,$$

where *ϕ*_*j*_ is the unit-amplitude radiation potential according to the definition $\phi_{R}=i\omega \sum_{j}\xi_{i}\phi_{j}$.

The contribution from the radiation potential is expressed in terms of the added mass and radiation damping, whose coefficients *m*_*ij*_ and *R*_*ij*_ are defined in the form

2.6 $$m_{ij}-\frac{i}{\omega }R_{ij}=\rho \iint_{S_{b}}\phi_{j}n_{i}\;dS=\rho \iint_{S_{b}}\phi_{j}\frac{\partial \phi_{i}}{\partial n}\;dS.$$

The hydrostatic restoring force coefficients, i.e. the change in the hydrostatic component of the generalized force *F*_*i*_ due to a unit displacement in mode *j*, are given as [16]

2.7 $$K_{ij}=\rho g\iint_{S_{b}}n_{j}(w_{i}+zD_{i})\;dS,$$

where *D*_*j*_ is the divergence of **S**_*j*_(**x**). For the additional mode (*j*=7),

2.8 $$D_{7}=\nabla \cdot {\text{S}}_{7}=0.$$

Lastly, the coefficients of the generalized mass matrix are given as

2.9 $$M_{ij}={∭}_{V}\rho_{m}{\text{S}}_{i}\cdot {\text{S}}_{j}\;dV,$$

where *ρ*_*m*_ is the density of the body, which is a function of **x**, and the integral is taken over the total volume of the body. We assume that the mass of the moving surface is zero. It then follows from (2.9) and (2.1) that

2.10 $$M_{37}=M_{73}=M_{77}=0,$$

while *M*_33_ is equal to the total mass *M* of the body, excluding the added mass.

## 3. Air-filled compressible bodies

### (a) Floating cylinder with downward-facing moving surface

**Figure 1.**
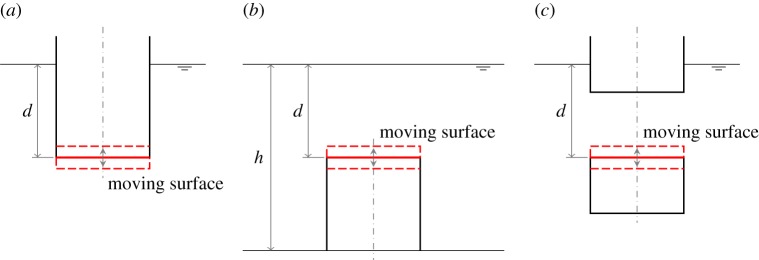
(*a*–*c*) Sketch of the air-filled compressible cylinders. For (*c*), the upper and lower cylinders move together as one body. For each cylinder, the moving surface is free to move up and down relative to the body. (Online version in colour.)

We consider first a compressible floating cylinder enclosing a volume of air, as shown in figure 1*a*. The total air volume may be greater or smaller than the submerged volume of the cylinder. The moving surface at the bottom is assumed to be a rigid horizontal surface, free to move up and down relative to the cylinder. There is then a pneumatic restoring force on the moving surface.

Using (2.1)–(2.3) and (2.8), it follows from (2.7) that

3.1 $$K_{37}=K_{73}=K_{77}=\rho g\iint_{S_{s}}n_{z}\;dS=\rho gS_{1},$$

where *S*_1_ is the projected area of the moving surface on the horizontal plane, while *K*_33_=*ρgS*_2_, where *S*_2_ is the water plane area of the cylinder. For a cylinder with a flat horizontal moving surface, *S*_1_ is just the area of the moving surface.

Let the equilibrium air volume and pressure in the compressible volume be *V*_10_ and *p*_0_=*p*_atm_+*ρgd*, where *d* is the equilibrium submergence of the moving surface (figure 1). Assuming a linearized isentropic air pressure–density relation, we have, for the compressible volume,

3.2 $$p_{1}=-\frac{\gamma p_{0}}{V_{10}}V_{1},$$

where *p*_1_ is the dynamic air pressure in the compressible volume and *V*_1_ is the volume change, which may be expressed as

3.3 $$V_{1}=-\xi_{7}S_{1}.$$

Substituting (3.3) into (3.2) and noting that the pneumatic force on the moving surface is pointing in the negative *z*-direction, we may write the pneumatic stiffness of the moving surface as

3.4 $$K_{p77}=S_{1}^{2}\frac{\gamma p_{0}}{V_{10}}.$$

Note that the pneumatic stiffness of the moving surface is linearly dependent on the ratio of the equilibrium pressure *p*_0_ to the compressible volume *V*_10_, and quadratically dependent on the area *S*_1_, whereas its hydrostatic stiffness is only linearly dependent on *S*_1_.

Putting all the terms together, we can write the coupled dynamic equations for the floating cylinder in the following matrix form:

3.5 $$\left\{-\omega^{2}\left[\begin{array}{cc} M+m_{33} & m_{37}\\ m_{73} & m_{77} \end{array}\right]+i\omega \left[\begin{array}{cc} R_{33} & R_{37}\\ R_{73} & R_{77} \end{array}\right]+\left[\begin{array}{cc} \rho gS_{2} & \rho gS_{1}\\ \rho gS_{1} & \rho gS_{1}+S_{1}^{2}\frac{\gamma p_{0}}{V_{10}} \end{array}\right]\right\}\left[\begin{array}{c} \xi_{3}\\ \xi_{7} \end{array}\right]=\left[\begin{array}{c} F_{e3}\\ F_{e7} \end{array}\right].$$

In accordance with (2.5) and (2.3), *F*_*e*7_ is the vertical component of the diffracted wave pressure integrated over the moving surface only, whereas *F*_*e*3_ is the vertical component of the diffracted wave pressure integrated over the entire wetted surface.

An estimate of the modified heave natural frequency of the cylinder due to compressibility of the submerged volume may be obtained based on quasi-static assumption. Under quasi-static assumption, the pressure change in the compressible volume is determined by hydrostatics only

3.6 $$p_{1}=-\rho g(\xi_{3}+\xi_{7}).$$

Combining (3.2), (3.3) and (3.6), we have the ratio of the relative displacement of the moving surface to the heave displacement of the cylinder

3.7 $$\frac{\xi_{7}}{\xi_{3}}\equiv r=-\frac{\rho g}{\gamma p_{0}S_{1}/V_{10}+\rho g}.$$

Noting that the change in buoyancy is given by

3.8 $$F_{b}=-\rho g(S_{2}\xi_{3}+S_{1}\xi_{7})=-\rho g\xi_{3}(S_{2}+S_{1}r),$$

we may therefore estimate the heave natural frequency of the compressible cylinder as

3.9 $$\omega_{n3}=\sqrt{\frac{\rho g(S_{2}+S_{1}r)}{M+m_{33}+m_{37}r}}.$$

As *p*_0_,*S*_1_ and *V*_10_ are positive, so −1≤*r*≤0 according to (3.7), and, assuming that *S*_1_≤*S*_2_, we have $0\leq \omega_{n3}\leq \sqrt{\rho gS_{2}/(M+m_{33})}$. The latter is just the heave natural frequency of a rigid cylinder. Note that *ω*_*n*3_→0 as $V_{10}\to \infty$.

### (b) Bottom-fixed cylinder with upward-facing moving surface

Next, we consider a bottom-fixed cylinder as shown in figure 1*b*. In this case, there is only one mode, i.e. the motion of the moving surface, defined by (2.1).

On the moving surface, the unit normal vector is pointing in the negative *z*-direction. The hydrostatic restoring coefficient is therefore negative and is given as (cf. (3.1))

3.10 $$K_{77}=-\rho gS_{1},$$

where *S*_1_ is the projected area of the moving surface on the horizontal plane. The volume change of the compressible volume is given as

3.11 $$V_{1}=\xi_{7}S_{1}.$$

Combining (3.11) with (3.2) and noting that the pneumatic force on the moving surface is pointing in the positive *z*-direction, the pneumatic stiffness of the moving surface is again as given in (3.4).

With *M*_77_=0, the dynamic equation for the moving surface can finally be written as

3.12 $$\left(-\omega^{2}m_{77}+i\omega R_{77}-\rho gS_{1}+S_{1}^{2}\frac{\gamma p_{0}}{V_{10}}\right)\xi_{7}=F_{e7},$$

and the natural frequency of the moving surface obtained as

3.13 $$\omega_{n7}=\sqrt{\frac{S_{1}(-\rho g+S_{1}\gamma p_{0}/V_{10})}{m_{77}}}.$$

As the total stiffness of the moving surface is a sum of the negative hydrostatic stiffness and the positive pneumatic stiffness, there is a possibility that the moving surface may be unstable. Stability of the moving surface requires that the sum of the pneumatic stiffness and the hydrostatic stiffness is positive, that is,

3.14 $$S_{1}>\frac{\rho gV_{10}}{\gamma p_{0}}.$$

This means that the area *S*_1_ has to be sufficiently large for a given volume *V*_10_ and pressure *p*_0_, or that *V*_10_ cannot be too large for a given area *S*_1_ and pressure *p*_0_.

Theoretically, according to (3.13), we can have $0\leq \omega_{n7}<\infty$. Note that *ω*_*n*7_→0 as *V*_10_→*γp*_0_*S*_1_/*ρg*, that is, the compressible volume does not have to be infinitely large to achieve *ω*_*n*7_→0, in contrast to the case of a floating cylinder with downward-facing moving surface, for which *ω*_*n*3_→0 as $V_{10}\to \infty$.

### (c) Floating cylinder with upward-facing moving surface

If we consider a floating cylinder as shown in figure 1*c*, since the unit normal vector on the moving surface is pointing in the negative *z*-direction, we have

3.15 $$K_{37}=K_{73}=K_{77}=-\rho gS_{1}$$

instead of (3.1). Also, equation (3.11) applies instead of (3.3). With (2.10) and (3.4) still applicable, the coupled dynamic equations for the cylinder can therefore be written in the form (cf. (3.5))

3.16 $$\begin{array}{r} \left\{-\omega^{2}\left[\begin{array}{cc} M+m_{33} & m_{37}\\ m_{73} & m_{77} \end{array}\right]+i\omega \left[\begin{array}{cc} R_{33} & R_{37}\\ R_{73} & R_{77} \end{array}\right]+\left[\begin{array}{cc} \rho gS_{2} & -\rho gS_{1}\\ -\rho gS_{1} & -\rho gS_{1}+S_{1}^{2}\frac{\gamma p_{0}}{V_{10}} \end{array}\right]\right\}\left[\begin{array}{c} \xi_{3}\\ \xi_{7} \end{array}\right]=\left[\begin{array}{c} F_{e3}\\ F_{e7} \end{array}\right]. \end{array}$$

The heave natural frequency of the cylinder may be estimated quasi-statically as in §3*a*. The ratio of the relative displacement of the moving surface to the heave displacement of the cylinder may be obtained by combining (3.2), (3.11) and (3.6) to give

3.17 $$\frac{\xi_{7}}{\xi_{3}}\equiv r=\frac{\rho g}{\gamma p_{0}S_{1}/V_{10}-\rho g}.$$

Stability of the moving surface requires the denominator of (3.17) to be positive, i.e. the condition (3.14). The change in buoyancy is given by

3.18 $$F_{b}=-\rho g(S_{2}\xi_{3}-S_{1}\xi_{7})=-\rho g\xi_{3}(S_{2}-S_{1}r).$$

The heave natural frequency of a compressible cylinder with upward-facing moving surface may therefore be estimated as

3.19 $$\omega_{n3}=\sqrt{\frac{\rho g(S_{2}-S_{1}r)}{M+m_{33}+m_{37}r}}.$$

Denoting the ratio *S*_1_/*S*_2_ as *r*_*S*_, stability of the cylinder requires that *r*<1/*r*_*S*_, or

3.20 $$S_{1}>\frac{\rho gV_{10}}{\gamma p_{0}}(1+r_{S}),$$

according to (3.17). This is a more stringent condition than (3.14). As 0≤*r*<1/*r*_*S*_, therefore $0\leq \omega_{n3}\leq \sqrt{\rho gS_{2}/(M+m_{33})}$. The latter is again the heave natural frequency of the cylinder if the moving surface were rigidly fixed to the cylinder. Note that *ω*_*n*3_→0 as *V*_10_→*γp*_0_*S*_1_/*ρg*(1+*r*_*S*_), in contrast to the floating cylinder with downward-facing moving surface, for which *ω*_*n*3_→0 as $V_{10}\to \infty$. This implies that the natural frequency *ω*_*n*3_ decreases more rapidly with the volume *V*_10_ for the cylinder with upward-facing moving surface than for the cylinder with downward-facing moving surface.

## 4. Air-filled compressible bodies as wave energy devices

### (a) Floating devices

**Figure 2.**
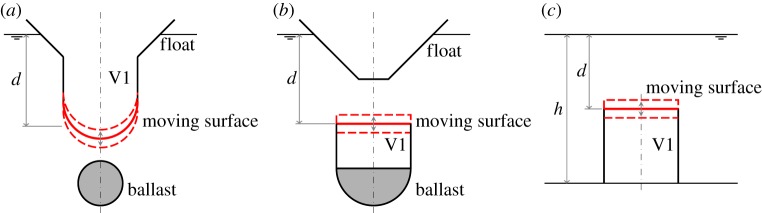
(*a*–*c*) Schematics of the air-filled compressible devices. The compressible volume V1, which may be greater or smaller than the volume enclosed above or beneath the moving surface, is connected via a turbine to a fixed volume V2, which may be contained in the float, or located onshore for the bottom-fixed device (*c*). For device (*a*), the ballast may alternatively be contained inside V1. Device (*c*) resembles that proposed by Budal & Falnes [9], §2.3. (Online version in colour.)

If the compressible volume of the cylinders in figure 1 is connected to a fixed volume via an air turbine for PTO, we have wave energy devices which absorb energy by pumping air between the two volumes as the moving surface oscillates relative to the cylinder, under wave action. Schematics of these devices are shown in figure 2, illustrating the general configurations. Note that, for the device in figure 2*a*, we have chosen the moving surface to be a hemispherical surface instead of a flat horizontal surface. In this case, the area *S*_1_ is the projected area of the hemisphere on the horizontal plane, and the equilibrium submergence *d* is measured from the mean free surface to a distance *a*/3 from the lowest point of the hemisphere, where *a* is the radius of the hemisphere.

As before, let us denote the equilibrium compressible volume as *V*_10_ and the equilibrium pressure in both the compressible and fixed volumes as *p*_0_=*ρgd*+*p*_atm_, while the equilibrium mass of air in each volume is denoted as *m*_10_ and *m*_20_. Assuming a linearized isentropic air pressure–density relation, for the fixed volume we have

4.1 $$p_{2}=\gamma p_{0}\frac{m_{2}}{m_{20}},$$

where *p*_2_ and *m*_2_ are, respectively, the dynamic air pressure and the change of air mass in the fixed volume. For the compressible volume, we have

4.2 $$\frac{V_{1}}{V_{10}}=\frac{m_{1}}{m_{10}}-\frac{p_{1}}{\gamma p_{0}},$$

where *V*_1_ is the volume change of the compressible volume, while *m*_1_ and *p*_1_ are, respectively, the change of air mass and the dynamic air pressure in the compressible volume.

If we assume that the flow through the turbine is governed by the following linear relationship:

4.3 $$i\omega m_{2}=-i\omega m_{1}=C(p_{1}-p_{2}),$$

where *C* is the mass flow through the turbine for a unit pressure difference, substituting (4.1) into (4.3) gives

4.4 $$m_{1}=-m_{2}=-m_{20}Dp_{1},\;\text{where}\;D=\frac{C}{\gamma p_{0}C+i\omega m_{20}}.$$

The volume change of the compressible volume is given by

4.5 $$V_{1}=\left\{ \begin{array}{ll} -\xi_{7}S_{1} & \text{for downward-facing moving surface}\\ \xi_{7}S_{1} & \text{for upward-facing moving surface}. \end{array}\right.$$

Substituting (4.4) into (4.2) and combining with (4.5), we have

4.6 $$\xi_{7}=\left\{ \begin{array}{ll} \frac{V_{10}}{S_{1}}\left(\frac{m_{20}}{m_{10}}D+\frac{1}{\gamma p_{0}}\right)p_{1} & \text{for downward-facing moving surface}\\ -\frac{V_{10}}{S_{1}}\left(\frac{m_{20}}{m_{10}}D+\frac{1}{\gamma p_{0}}\right)p_{1} & \text{for upward-facing moving surface}. \end{array}\right.$$

Let *L* be a complex non-dimensional quantity defined as

4.7 $$L\equiv \frac{S_{1}}{\rho gV_{10}((m_{20}/m_{10})D+1/\gamma p_{0})}.$$

The pneumatic force on the moving surface due to a unit relative displacement of the same is therefore given as −*S*_1_*ρgL*, regardless of whether the moving surface is downward- or upward-facing. As *L* is complex, this force on the moving surface may be decomposed into a real part which is proportional to its displacement, and an imaginary part which is proportional to its velocity. The pneumatic stiffness and damping coefficients *K*_*p*77_ and *R*_*p*77_ may thus be expressed as

4.8 Kp77=ρgS1 Re{L}andRp77=ρgS1ω Im{L}.

Note that both coefficients depend on the wave frequency *ω*.

For the floating device, the coupled dynamic equations may therefore be written in the form

4.9 {−ω2[M+m33m37m73m77]+iω[R33R37R73R77+ρgS1ω Im{L}]+[ρgS2±ρgS1±ρgS1±ρgS1+ρgS1 Re{L}]}[ξ3ξ7]=[Fe3Fe7],

where + and − in ± correspond to downward-facing moving surface and upward-facing moving surface, respectively. It is clearly seen that the presence of the turbine modifies both the stiffness and damping of the moving surface. When the turbine is blocked, i.e. when *C*=0, equation (4.9) reduces to (3.5) if the moving surface is downward-facing, or to (3.16) if the moving surface is upward-facing.

Equation (4.9) can be solved for the displacements *ξ*_3_ and *ξ*_7_, and, upon finding *p*_1_ and *p*_2_ from, for example (4.6), (4.4) and (4.3), the mean absorbed power in regular waves may be obtained from

4.10 $$P=\frac{C}{2\rho_{\mathrm{air}}}|p_{2}-p_{1}{|}^{2},$$

where *ρ*_air_ is the equlibrium air density in both volumes.

### (b) Bottom-fixed device

For the bottom-fixed device (figure 2*c*), which has an upward-facing moving surface, equations (4.1)–(4.4), (4.7) and (4.8), as well as the second lines of equations (4.5) and (4.6), apply. The dynamic equation may thus be written as (cf. (4.9) and (3.12))

4.11 [−ω2m77+iω(R77+ρgS1ω Im{L})−ρgS1+ρgS1 Re{L}]ξ7=Fe7.

The mean absorbed power may be obtained, as in the floating device, using (4.10).

## 5. Air-filled compressible devices with water columns

### (a) Bottom-fixed device with a water column

**Figure 3.**
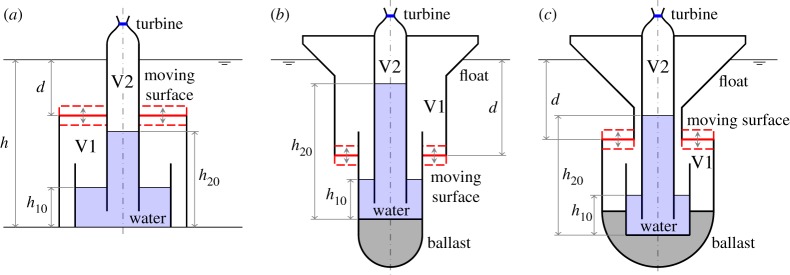
(*a*–*c*) Schematics of the compressible devices with water columns. Part of V1 and the water column may alternatively be located above water or onshore. (Online version in colour.)

If the compressible volume of the bottom-fixed cylinder in figure 1*b* is connected to a water column, and the air volume above the water column is fitted with an air turbine open to the atmosphere, we have another device variation. The water column replaces the fixed volume in the devices described in §4, in providing the required restoring force on the compressible volume. A possible arrangement is shown in figure 3*a*. Under wave excitations, the moving surface and the water column, which are coupled via the compressible volume V1, oscillate, pumping air into and out of the volume V2 via the turbine.

Let us denote the equilibrium volumes in V1 and V2 as *V*_10_ and *V*_20_, respectively. The equilibrium pressure in V1 is *p*_0_=*ρgd*+*p*_atm_, where *d* is the submergence of the moving surface, while the equilibrium pressure in V2 is *p*_atm_. The equilibrium water column levels are related as *h*_20_=*h*_10_+*d*. Assuming an isentropic air pressure–density relation, for volume V1, relation (3.2) holds, while for volume V2,

5.1 $$\frac{V_{2}}{V_{20}}=\frac{m_{2}}{m_{20}}-\frac{p_{2}}{\gamma p_{\mathrm{atm}}}.$$

The flow through the turbine is idealized according to the following linear relationship:

5.2 $$-i\omega m_{2}=Cp_{2},$$

where *C* is the mass flow through the turbine for a unit pressure difference.

Under the assumption that water is incompressible, the amplitudes of the outer and inner water column levels, *h*_1_ and *h*_2_, are related through the cross-sectional areas *S*_*t*1_ and *S*_*t*2_ according to *S*_*t*1_*h*_1_=−*S*_*t*2_*h*_2_. The volume amplitudes in V1 and V2 are given as

5.3 $$V_{1}=-h_{1}S_{t1}+\xi_{7}S_{1}=h_{2}S_{t2}+\xi_{7}S_{1}$$

and

5.4 $$V_{2}=-h_{2}S_{t2}=h_{1}S_{t1}.$$

Employing the lumped-parameter approach, we may write the equation of motion for the water column, assuming no losses, as

5.5 $$p_{1}-p_{2}=-\omega^{2}\rho h_{2}\left[d+h_{10}\left(1+\frac{S_{t2}}{S_{t1}}\right)\right]+\rho gh_{2}\left(1+\frac{S_{t2}}{S_{t1}}\right).$$

On the right-hand side, the first and second terms are the inertia and the restoring terms.

Using (3.2), and (5.1)–(5.4), we may eliminate *p*_1_ and *p*_2_ in (5.5) to obtain an equation of motion for the water column in terms of the variables *h*_2_ and *ξ*_7_. We can then write the coupled equations of motion for the moving surface and the water column in the following matrix form:

5.6 {−ω2[m7700ρ(d+h10f)]+iω[R7700ρgω Im{L}]+[−ρgS1+S12γp0V10S1St2γp0V10S1γp0V10ρgf+St2γp0V10+ρg Re{L}]}[ξ7h2]=[Fe70],

where the non-dimensional quantities *f* and *L* are defined as

5.7 $$f\equiv 1+\frac{S_{t2}}{S_{t1}}\;\mathrm{and}\;L\equiv \frac{S_{t2}}{\rho gV_{20}(C/i\omega m_{20}+1/\gamma p_{\mathrm{atm}})}.$$

Equation (5.6) can be solved for *ξ*_7_ and *h*_2_, and upon finding *p*_2_ using (5.1), (5.2) and (5.4), we may obtain the mean absorbed power in regular waves as

5.8 $$P=\frac{C}{2\rho_{\mathrm{air}}}|p_{2}{|}^{2},$$

where *ρ*_air_ is the atmospheric air density.

The water column resonance when the moving surface is fixed to the body may be derived from (5.5) as

5.9 $$\omega_{0}=\sqrt{\frac{g}{l}},$$

where

5.10 $$l=\left\{ \begin{array}{ll} \frac{d+h_{10}f}{f+S_{t2}/S_{p1}+S_{t2}/S_{p2}} & \text{when }C=0\;\text{(V2 is closed)}\\ \frac{d+h_{10}f}{f+S_{t2}/S_{p1}} & \text{when V2 is completely open to the atmosphere}, \end{array}\right.$$

with

5.11 $$S_{p1}\equiv \frac{V_{10}}{\gamma (d+p_{\mathrm{atm}}/\rho g)}\;\mathrm{and}\;S_{p2}\equiv \frac{\rho gV_{20}}{\gamma p_{\mathrm{atm}}}.$$

### (b) Floating devices with a water column

The water column idea can be applied to floating devices as well. In addition to the components of the bottom-fixed device (§5*a*), the floating device has a float at the top and an amount of ballast to balance the buoyancy force (figure 3*b*,*c*).

If the displacement of the moving surface and the levels of the water column are defined relative to the float, then equations (3.2), (5.1), (5.2) and (5.4) apply without change. The volume amplitude in V1 is given as

5.12 $$V_{1}=\left\{ \begin{array}{ll} -h_{1}S_{t1}-\xi_{7}S_{1}=h_{2}S_{t2}-\xi_{7}S_{1} & \text{for downward-facing moving surface}\\ -h_{1}S_{t1}+\xi_{7}S_{1}=h_{2}S_{t2}+\xi_{7}S_{1} & \text{for upward-facing moving surface.} \end{array}\right.$$

Equation (5.5) is modified to include a coupling from the acceleration of the float

5.13 $$p_{1}-p_{2}=-\omega^{2}\rho h_{2}\left[d+h_{10}\left(1+\frac{S_{t2}}{S_{t1}}\right)\right]+\rho gh_{2}\left(1+\frac{S_{t2}}{S_{t1}}\right)-\omega^{2}\rho d\xi_{3}.$$

In addition, momentum conservation dictates that, due to the acceleration of the water column, the float experiences a vertical force which is given as *ω*^2^*h*_2_*ρdS*_*t*2_.

Proceeding as in the case of the bottom-fixed device, we may then write the coupled equations of motion for the float, the moving surface and the water column as follows:

5.14 {−ω2[M+m33m37ρdSt2m73m770ρd0ρ(d+h10f)]+iω[R33R370R73R77000ρgω Im{L}]+[ρgS2±ρgS10±ρgS1±ρgS1+S12γp0V10∓S1St2γp0V100∓S1γp0V10ρgf+St2γp0V10+ρg Re{L}]}[ξ3ξ7h2]=[Fe3Fe70],

where + and − in ±, as well as − and + in ∓, correspond to downward-facing moving surface and upward-facing moving surface, respectively. The non-dimensional quantities *f* and *L* are as defined in (5.7). As in the bottom-fixed device, the mean absorbed power may be obtained using (5.8).

## 6. Results and discussions

The hydrodynamic coefficients, i.e. the added mass, radiation damping and wave exciting force coefficients, for all the geometries considered in this paper are computed using a three-dimensional radiation/diffraction program [17], which is based on linear potential theory. For the floating configurations, the water depth is assumed to be infinite. It should be noted that the following numerical results are obtained using linear models, and losses have not been taken into account. As such, they should be regarded as optimistic estimates.

### (a) Compressible bodies

**Figure 4.**
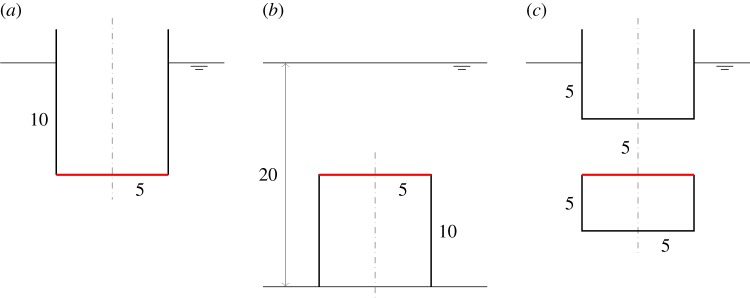
(*a*–*c*) Dimensions (in metres) of the compressible cylinders used in the numerical calculations. The three configurations have the same equilibrium submerged volume of 785 m^3^. (Online version in colour.)

**Figure 5.**
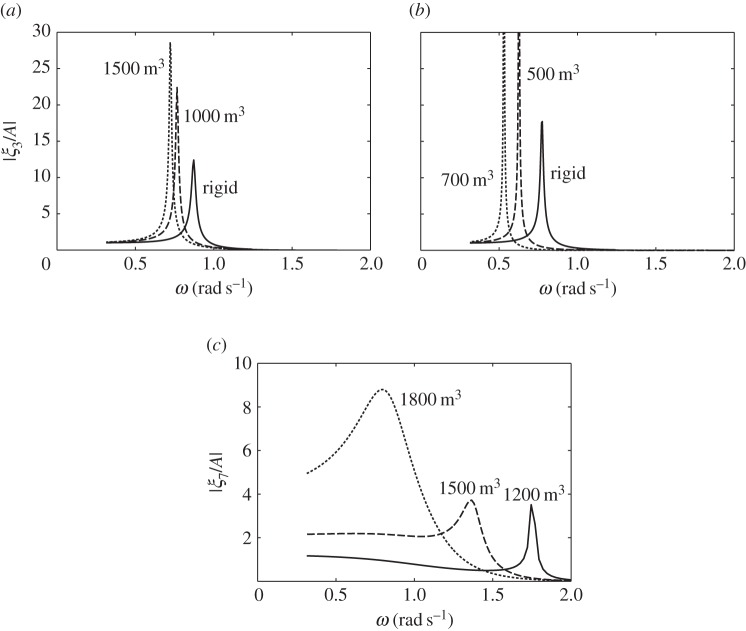
Heave displacements, per unit incident wave amplitude, of (*a*) the cylinder in figure 4*a* and (*b*) the cylinder in figure 4*c* and (*c*) moving surface displacements, per unit incident wave amplitude, of the bottom-fixed cylinder in figure 4*b*, for various *V*_10_.

The dimensions of the compressible cylinders chosen for the numerical calculations are shown in figure 4. Figure 5*a*,*b* shows the calculated heave displacements, per unit incident wave amplitude *A*, of the floating cylinders with downward- and upward-facing moving surfaces, for various equilibrium compressible volume *V*_10_. For the floating cylinder with a 5-m-radius upward-facing moving surface, stability condition (3.20) requires that *V*_10_ cannot be greater than 1104 m^3^. It is shown from both figures that compressibility lowers the heave natural frequencies of the cylinders in comparison with the rigid cylinders. For the floating cylinder with an upward-facing moving surface, we do not require as much *V*_10_ as that of the cylinder with a downward-facing moving surface to obtain the same reduction in natural frequency. This observation agrees with our expectation in §3, namely that the heave natural frequency decreases more rapidly with *V*_10_ for the cylinder with upward-facing moving surface than for the cylinder with downward-facing moving surface.

The calculated moving surface displacements of the bottom-fixed cylinder for various *V*_10_ are shown in figure 5*c*. For the bottom-fixed cylinder with a 5-m-radius moving surface, stability condition (3.14) requires that *V*_10_ cannot be greater than 2208 m^3^. The natural frequency of the moving surface is shown to be sensitive to the variation of *V*_10_. A broad-banded resonance covering typical ocean wave frequencies can be obtained for a sufficiently large value of *V*_10_. The value of *V*_10_ also determines the displacement of the moving surface at the zero-frequency limit, which may be written as (cf. (3.12))

6.1 $$\lim_{\omega \to 0}\xi_{7}=\frac{\rho gS_{1}A}{S_{1}^{2}\gamma p_{0}/V_{10}-\rho gS_{1}},$$

where *ρgS*_1_*A* is the wave exciting force on the moving surface as *ω*→0. equation (6.1) gives $\lim_{\omega \to 0}\xi_{7}/A$ values of 1.2, 2.1 and 4.4 corresponding to *V*_10_ of 1200, 1500 and 1800 m^3^, in agreement with figure 5*c*.

**Figure 6.**
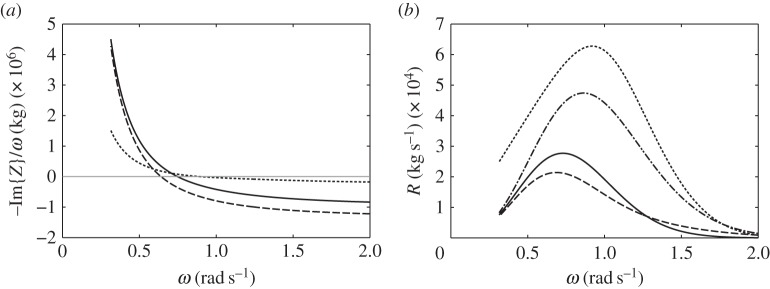
(*a*) Plots of −Im{*Z*}/*ω*, where *Z* is the impedance of the system, for the floating cylinder with a downward-facing moving surface and *V*_10_= 1000 m^3^ (solid line); the floating cylinder with an upward-facing moving surface and *V*_10_=500 m^3^ (dashed line); the bottom-fixed cylinder with an upward-facing moving surface and *V*_10_=1800 m^3^ (dotted line). Resonance is achieved when Im{*Z*}=0. (*b*) Radiation damping coefficients of the compressible bodies: *R*_33_=*R*_77_ for the floating cylinder with a downward-facing moving surface (solid line); *R*_33_ for the floating cylinder with an upward-facing moving surface (dashed line); *R*_77_ for the floating cylinder with an upward-facing moving surface (dashed dotted line); *R*_77_ for the bottom-fixed cylinder with an upward-facing moving surface (dotted line).

The broad-bandedness of the displacement curve for the bottom-fixed cylinder, as seen in figure 5, relative to those for the floating cylinders may be explained by looking at figure 6. In figure 6*a*, we plot the imaginary part of the system impedance *Z* for each of the three cylinders with representative *V*_10_ values. For the floating cylinders, we reduce the system impedance from a matrix to a scalar using the quasi-static approximation described earlier. Resonance happens when Im{*Z*}=0. It is seen that, for the bottom-fixed cylinder, Im{*Z*} is much closer to zero over a wide range of frequencies, compared with that for the floating cylinders.

Figure 6*b* shows that the moving surface of the bottom-fixed cylinder has the highest amount of radiation damping. As the radiation damping coefficient corresponds to the amount of energy that the body is able to radiate, it is notable that an upward-facing moving surface is a better wave radiator than a downward-facing moving surface of the same size and submergence. Furthermore, in the case of the floating cylinders, the total mass is the displaced mass plus the added mass of the body. For the bottom-fixed cylinder, however, the total mass is just the added mass of the moving surface, as the rest of the cylinder is stationary. As resonance bandwidth is determined by the ratio of the system damping to the system mass (e.g. [18], §§2.1 and 3.5), the high radiation damping and low mass associated with the moving surface of the bottom-fixed cylinder explain its broad-bandedness.

### (b) Devices without water columns

**Figure 7.**
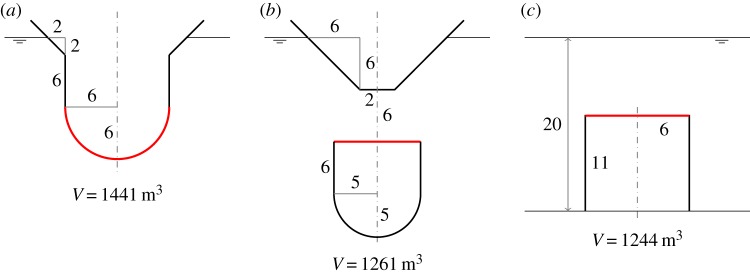
(*a*–*c*) Dimensions (in metres) of the devices without water columns. The displaced volume *V* of each device is indicated. (Online version in colour.)

**Figure 8.**
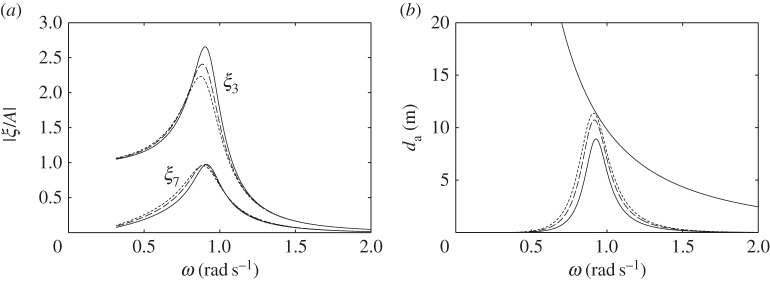
(*a*) Displacements, per unit incident wave amplitude, and (*b*) absorption widths of the device in figure 7*a*, for *C*=0.02 ms, *V*_10_=1000 m^3^ and various *V*_20_: *V*_20_=2000 m^3^ (solid line); *V*_20_=3000 m^3^ (dashed line); *V*_20_=4000 m^3^ (dotted line). The descending line in (*b*) is the theoretical maximum λ/2*π*.

For the devices without water columns, the dimensions used in the numerical calculations are shown in figure 7. We consider first the floating device with a downward-facing moving surface (figure 7*a*). Figure 8*a* shows that the heave of the body, normalized with the incident wave amplitude, tends to the value of one at low frequencies, whereas the normalized relative displacement of the moving surface tends to zero at low frequencies. This is as expected, since, for long waves, the whole device moves together with the wave.

As in the compressible bodies, to achieve the same reduction in natural frequency, a floating device with a downward-facing moving surface needs a larger compressible volume *V*_10_ than a floating device with an upward-facing moving surface does. Moreover, as figure 8*b* shows, the fixed volume *V*_20_ of a floating device with a downward-facing moving surface has to be sufficiently large to generate adequate flow through the turbine. Otherwise, the power absorption of the device is below the theoretical maximum, even at resonance. Thus, although the displaced volume of the device can be small, a large storage is required to house *V*_20_.

**Figure 9.**
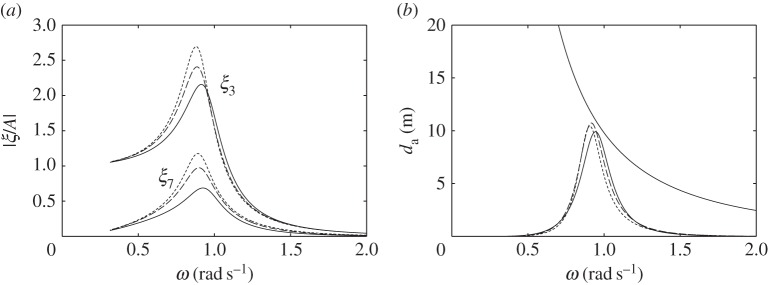
(*a*) Displacements, per unit incident wave amplitude, and (*b*) absorption widths of the device in figure 7*a*, for *V*_10_=1000 m^3^, *V*_20_= 3000 m^3^ and various turbine coefficients *C*: *C*=0.01 ms (solid line); *C*=0.02 ms (dashed line); *C*=0.03 ms (dotted line).

Figure 9 shows that increasing the turbine coefficient *C* increases the displacement peaks, as high *C* means low damping. Varying *C* shifts the peak of the absorption width curve within a range determined by *V*_20_ when *V*_10_ is kept constant. This is more clearly seen below in the case of a floating device with an upward-facing moving surface.

**Figure 10.**
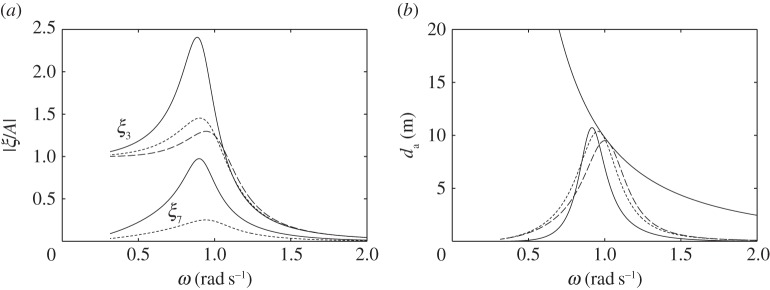
(*a*) Displacements, per unit incident wave amplitude, and (*b*) absorption widths of the device in figure 7*a*, for *C*=0.02 ms, *V*_10_= 1000 m^3^ and *V*_20_=3000 m^3^ (solid line), compared with those of a completely rigid device with equal dimensions taking off power by a linear damper (dashed line) and of a compressible device with equal dimensions taking off power by a linear damper (dotted line).

In figure 10, we compare the performance of the device in figure 7*a* with those of a completely rigid device and a compressible device both having the same dimensions as the device in figure 7*a*, but where, for each, power is taken from the body heave via a linear damper reacting against a fixed reference. The PTO damping coefficient is chosen to be 3×10^5^ kg s^−1^, which is approximately equal to the heave radiation damping coefficient of the rigid body at resonance. It is evident from figure 10*b* that the peak of the absorption width curve for the rigid device is at a frequency higher than those for the compressible devices, demonstrating the positive effect of a compressible volume in lowering the resonance frequency. The bandwidth of the device in figure 7*a* is however narrower than those of the devices with linear PTO dampers. Note, however, that the latter two devices are not self-reacting, but require a fixed reference for the PTO damper to react against.

**Figure 11.**
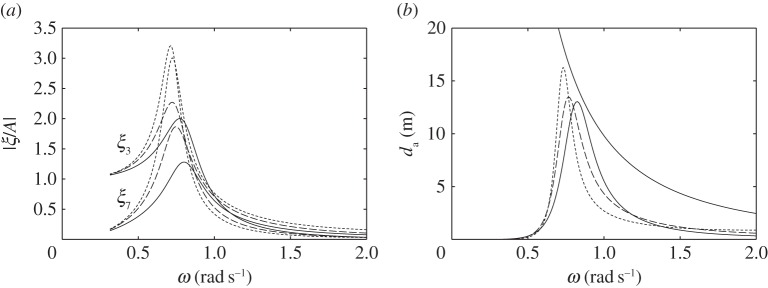
(*a*) Displacements, per unit incident wave amplitude, and (*b*) absorption widths of the device in figure 7*b*, for *V*_10_=600 m^3^, *V*_20_= 500 m^3^ and various turbine coefficients *C*: *C*=0.003 ms (solid line); *C*=0.006 ms (dashed line); *C*=0.012 ms (dotted line).

For the floating device with an upward-facing moving surface as shown in figure 7*b*, we do not need as much compressible volume *V*_10_ as the floating device with a downward-facing moving surface does. Also, the required fixed volume *V*_20_ is much less, and, thus, storage of *V*_20_ is not a problem. Figure 11 shows that varying the turbine coefficient *C* has the effect of shifting the peaks of the displacement and the absorption width curves within a determined range, as previously observed for the device with a downward-facing moving surface (cf. figure 9).

**Figure 12.**
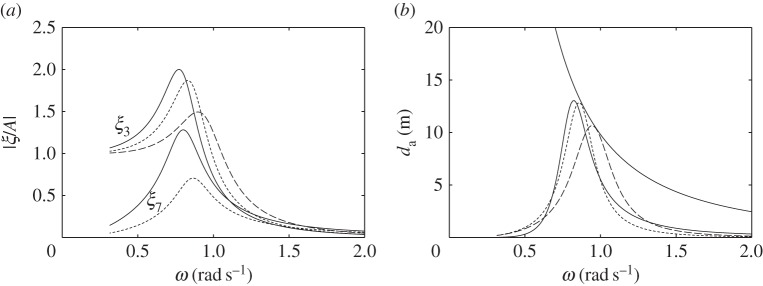
(*a*) Displacements, per unit incident wave amplitude, and (*b*) absorption widths of the device in figure 7*b*, for *C*=0.003 ms, *V*_10_= 600 m^3^ and *V*_20_=500 m^3^ (solid line), compared with those of a completely rigid device with equal dimensions taking off power by a linear damper (dashed line) and of a compressible device with equal dimensions taking off power by a linear damper (dotted line).

The performance of the device in figure 7*b* is compared in figure 12 with those of a completely rigid device and a compressible device both having the same dimensions as the device in figure 7*b*, but where, for each, power is taken from the body heave by a linear damper, whose coefficient is chosen to be 3×10^5^ kg s^−1^. This value is approximately equal to the heave radiation damping coefficient of the rigid body at resonance. The bandwidth of the device in figure 7*b* is shown in figure 12*b* to be comparable to those of the devices with linear PTO dampers. As the device in figure 7*b* is self-reacting rather than requiring a fixed reference, it is arguably a better device than those with linear PTO dampers and equal dimensions.

Comparing the dashed curve in figure 12*b* with that in figure 10*b*, i.e. the curves corresponding to the rigid devices with dimensions given in figure 7*b*,*a*, respectively, we may note that the peak in figure 12*b* is at a lower frequency than that in figure 10*b*, although the displaced mass of the device in figure 7*b* is smaller than that of the device in figure 7*a* and the waterplane area of the two devices are the same. This is because the heave added mass of the device in figure 7*b* is higher than that of the device in figure 7*a*, due to the presence of the gap in the former device between the upper and lower parts of the body.

Before we move on to the bottom-fixed device, it is important to note that, for a floating body with an upward-facing moving surface, the wave exciting forces on the moving surface *F*_*e*7_ and on the body *F*_*e*3_ are in opposite phases. By a well-known reciprocity relation between radiation damping and exciting force [19], the off-diagonal radiation damping coefficients *R*_37_=*R*_73_ are therefore negative. On the other hand, for a floating body with a downward-facing moving surface, the exciting forces *F*_*e*7_ and *F*_*e*3_ are in phase. The radiation damping coefficients *R*_37_=*R*_73_ are therefore positive. Furthermore, for a floating body with a downward-facing moving surface, the moving surface moves approximately in antiphase with the body. As the body moves down, the moving surface moves up relative to the body. On the other hand, for a floating body with an upward-facing moving surface, when the resonance frequency of the moving surface is much higher than the heave resonance frequency of the body, as considered here, the moving surface moves approximately in phase with the body. As the body moves down, the moving surface also moves down relative to the body. It follows that, for the same heave amplitude, the total wave energy radiated by a compressible heaving body with a relatively stiff moving surface is always less than the total wave energy radiated by a completely rigid body of the same dimensions, irrespective of the moving surface orientation.

**Figure 13.**
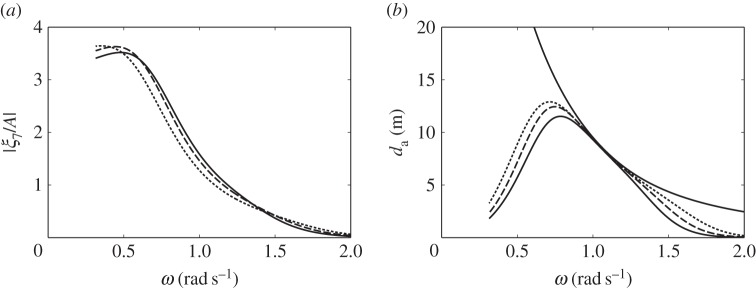
(*a*) Moving surface displacements, per unit incident wave amplitude, and (*b*) absorption widths of the device in figure 7*c*, for *C*=0.04 ms, *V*_10_= 1000 m^3^ and *V*_20_=1300 m^3^ (solid line), compared with those of a device with equal radius and *V*_10_ but with *d*=8 m, *C*=0.03 ms and *V*_20_=1200 m^3^ (dashed line) and a device with equal radius and *V*_10_ but with *d*=7 m, *C*=0.02 ms and *V*_20_=1100 m^3^ (dotted line).

In figure 13, we plot the moving surface displacement and absorption width of the bottom-fixed device with dimensions as shown in figure 7*c*, together with those of the devices having smaller moving surface submergences. A notable feature of the bottom-fixed device is its broad-banded resonance. As discussed in §6*a*, this broad-bandedness is due to the high ratio of radiation damping to added mass corresponding to the moving surface. The total air volume *V*_10_+*V*_20_ has to be sufficiently large to achieve resonance at typical ocean wave frequencies, as discussed previously. The individual values of *V*_10_ and *V*_20_ do not matter, as similar performance can be obtained for various combinations of *V*_10_ and *V*_20_ which give the same combined total volume, provided the turbine coefficient *C* is varied accordingly. This offers more design flexibility. The total air volume also determines the moving surface displacement at the zero frequency limit, whose value is given as in (6.1), but with *V*_10_ replaced by *V*_10_+*V*_20_. This gives $\lim_{\omega \to 0}\xi_{7}/A$ of 3.2, 3.3 and 3.5, corresponding to figure 13*a*.

The required air volume is somewhat reduced when the submergence of the moving surface is smaller, as the equilibrium pressure *p*_0_ is reduced as submergence is decreased. Moreover, having the equilibrium position of the moving surface higher up increases its radiation damping, which in turn results in a broader resonance bandwidth, as figure 13*b* shows.

### (c) Devices with water columns

**Figure 14.**
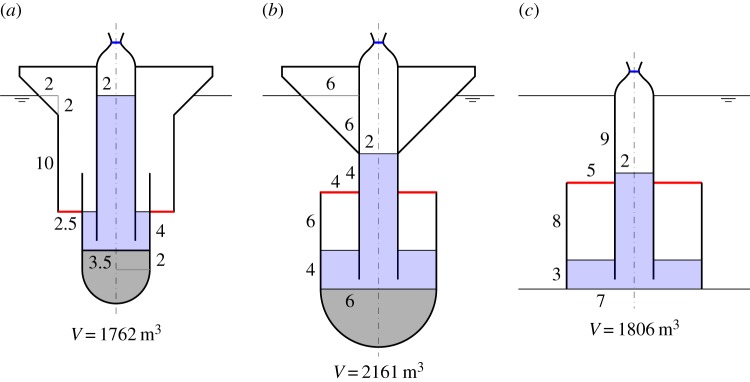
(*a*–*c*) Dimensions (in metres) of the devices with water columns. The displaced volume *V* of each device is indicated. (Online version in colour.)

**Figure 15.**
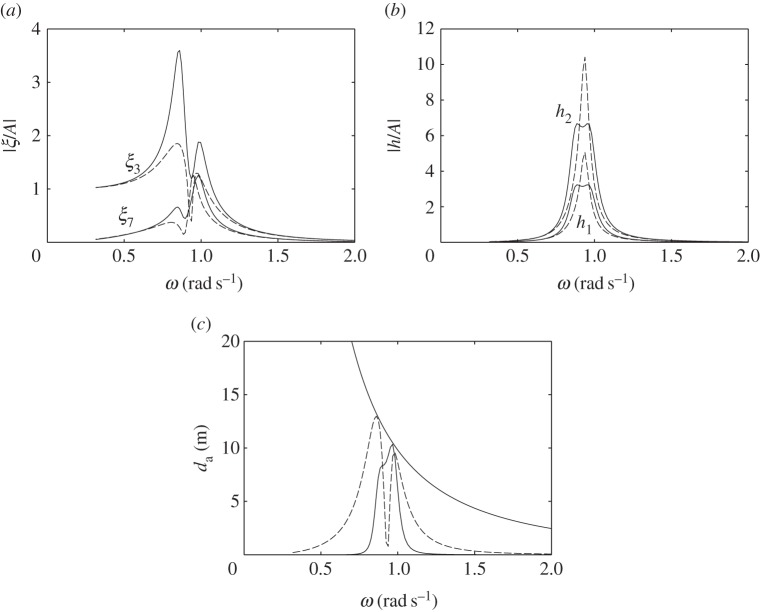
(*a*) Displacements and (*b*) water column amplitudes per unit incident wave amplitude, and (*c*) absorption widths of the device in figure 14*a*, for *C*=0.015 ms, *V*_10_=1500 m^3^ and *V*_20_=250 m^3^ (solid line), compared with those of a device with equal dimensions but taking off power by a linear damper from the body heave (dashed line).

For the devices with water columns, the dimensions used in the numerical calculations are shown in figure 14. We consider first the floating device with a downward-facing moving surface as shown in figure 14*a*. The water column introduces two peaks in both the displacement and the absorption width curves (figure 15). The frequency of the trough corresponds approximately to the water column resonance frequency when the moving surface is fixed to the body, as given in (5.9)–(5.11). Although we do not require as much air volume as that required by the device without the water column (figure 7*a*) to reach the theoretical maximum absorbed power, the power absorption bandwidth is very narrow. Increasing *V*_10_ may widen the bandwidth, but only slightly. The performance of the device in figure 14*a* appears to be relatively poor compared with the other devices considered so far.

In figure 15, we also compare the response of this device with that of a device of equal dimensions but with the turbine removed, i.e. with *V*_2_ completely open to the atmosphere, and where power is extracted from the body heave by a linear damper reacting against a fixed reference. The absorbed power has a minimum at a frequency corresponding to the resonance frequency of the water column. This is as expected, since, at this frequency, the water column motion is amplified, while the body becomes relatively stationary. Apart from this minimum, the absorbed power bandwidth is broader, as it corresponds to the bandwidth of the body heave, which is broader than that of the water column.

**Figure 16.**
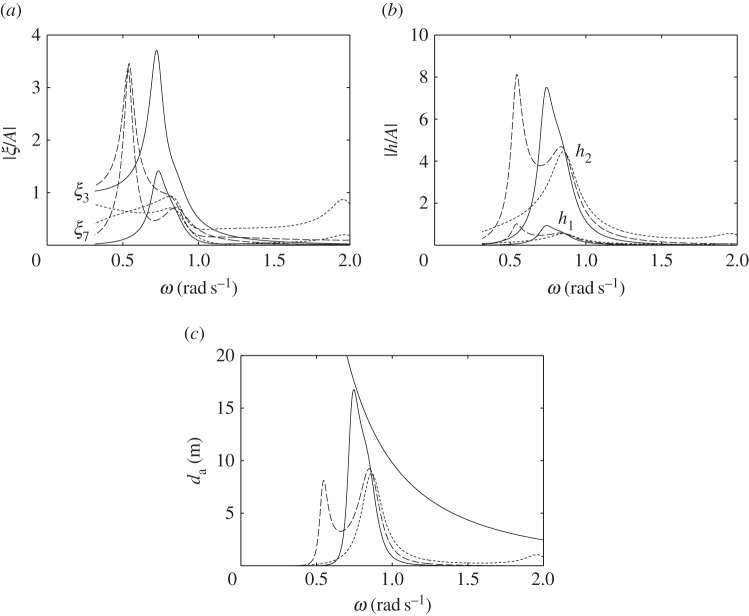
(*a*) Displacements and (*b*) water column amplitudes per unit incident wave amplitude, and (*c*) absorption widths of the device in figure 14*b*, for *C*= 0.005 ms, *V*_20_=250 m^3^ and various *V*_10_: *V*_10_=300 m^3^ (solid line); *V*_10_=1000 m^3^ (dashed line); *V*_10_=1700 m^3^ (dotted line).

For the floating device with an upward-facing moving surface (figure 14*b*), an interesting effect is observed when the equilibrium compressible volume *V*_10_ is varied (figure 16). For a sufficiently small *V*_10_, better performance is obtained relative to that of the device with a downward-facing moving surface (figure 14*a*). When *V*_10_ is increased, the absorption width curve widens towards lower frequencies, but at the same time a trough is created which also widens as *V*_10_ is increased. Further increasing *V*_10_ removes the low-frequency peak and adds a high-frequency peak to the absorption width curve. The peak that remains at the same frequency corresponds to the heave natural frequency of the body. This behaviour may be explained by recalling that the water column resonance frequency depends on the volume *V*_10_, as shown in equations (5.9)–(5.11). The high-frequency peak observed when *V*_10_ is further increased corresponds to the resonance frequency of the moving surface.

**Figure 17.**
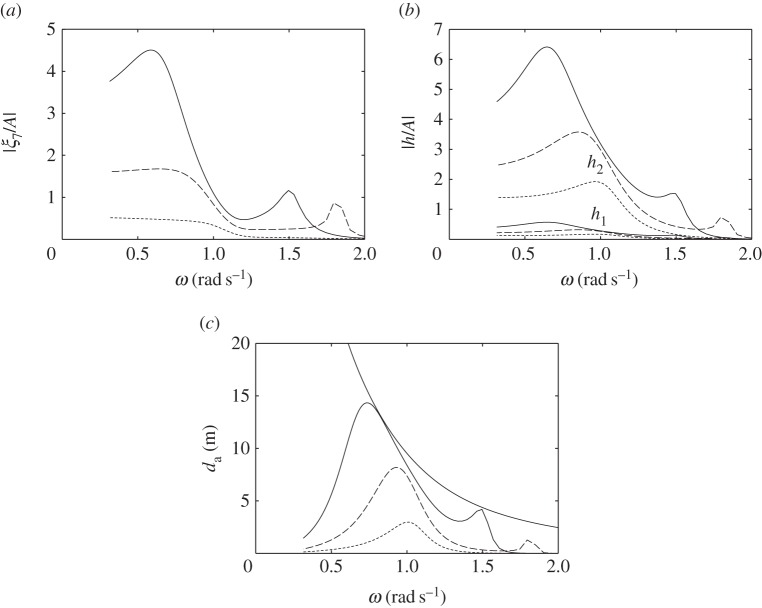
(*a*) Moving surface displacement and (*b*) water column amplitudes per unit incident wave amplitude, and (*c*) absorption width of the device in figure 14*c*, for *C*=0.002 ms, *V*_20_=250 m^3^ and various $V_{10}\;:\;V_{10}=2600\;m^{3}$ (solid line); *V*_10_=2000 m^3^ (dashed line); *V*_10_=1000 m^3^ (dotted line).

The response of the bottom-fixed device with a water column (figure 14*c*) for various values of compressible volume *V*_10_ is shown in figure 17. A broad-banded power absorption covering typical ocean wave frequencies is obtained with a sufficiently large *V*_10_. Reducing *V*_10_ decreases the displacements of the device, but also decreases the amount of absorbed power. The water column introduces a trough in the power absorption curve, and it appears that the water column does not offer any clear advantages relative to a device without a water column (cf. figure 13*b*).

The poor performance of the floating devices with water columns may be explained by comparing them with the bottom-fixed counterpart. The latter has two modes: the moving surface displacement and the water column displacement. The two are coupled through the compressible volume *V*_1_ (see (5.6)). With a sufficiently large *V*_10_, the moving surface has a broad-banded resonance, which is passed on to the water column in the manner of two coupled oscillators where only the main mass is excited. The main mass in this case is the moving surface. On the other hand, the floating devices with water columns have three modes: the heave of the body, the displacement of the moving surface and the displacement of the water column. The dynamic interactions between the three is more complex. For the configurations considered here, the moving surface is relatively stiff, i.e. its resonance frequency is much higher than the heave resonance frequency of the body. In this case, the water column is excited mainly by the body heave instead of the moving surface (see (5.14)). It is therefore necessary to match the resonance frequency of the water column to the heave resonance frequency of the body. As we have just observed, however, the resulting power absorption bandwidth is narrow. When *V*_10_ is increased (figure 16), the water column resonance frequency moves away from the heave natural frequency of the body, and it results in two separate narrow peaks.

### (d) Further remarks

For a floating device with a downward-facing moving surface, with or without a water column, we may increase the air volume *V*_10_ to lower the resonance frequency of the moving surface, without any concerns about stability. However, it is not possible to lower the resonance frequency of the moving surface below a certain value, which is determined by the area of the moving surface. The resonance frequency of a downward-facing moving surface therefore remains much higher than the heave resonance frequency of the body. On the other hand, for a floating device with an upward-facing moving surface, it might be possible for the resonance frequency of the moving surface to approach typical wave frequencies by increasing *V*_10_ within the limits defined by the stability requirement. When the resonance frequency of the moving surface approaches that of the body, however, instead of moving in phase relative to the body, the moving surface begins to move in antiphase relative to the body. This means that, as the body moves up, the moving surface moves down, i.e. buoyancy drops, and so the upward motion of the body is reduced. While the moving surface motion itself might be amplified, the combined wave radiation of the two modes when both are in resonance is generally less than that when only one mode is in resonance. As we need optimum wave radiation for wave absorption, it follows that, for the floating devices with upward-facing moving surface, tuning the moving surface by increasing *V*_10_ such that its resonance frequency is close to that of the body heave is detrimental to the power absorption of the device.

One way to improve the performance of the floating devices with upward-facing moving surface involves tuning the moving surface, by increasing the compressible volume *V*_10_, to resonate at typical wave frequencies, while tuning the heave resonance of the body away from those frequencies. The body then serves as a relatively stationary reference for the moving surface to do work, and the moving surface becomes the main wave radiator. In this case, the device behaves almost like the bottom-fixed device. In [20], the approach was to increase the heave added mass of the body by modifying its geometry, such that its resonance frequency is lowered. Another possible approach is to increase the heave stiffness of the body, e.g. by tethering it to the sea bottom, so that its resonance frequency is increased. Both approaches, provided the air volume *V*_10_ is sufficiently large, would result in a broad absorbed power bandwidth. The latter approach would result in device dynamics more closely resembling the bottom-fixed device, so we expect a slightly broader bandwidth than what we would obtain from the former.

## 7. Conclusion

A heaving wave energy device should have a large waterplane area for it to radiate well. However, a large waterplane area means high stiffness, and therefore the displaced mass has to be large for the device to resonate with the waves. This is true for a rigid device. A heaving body with a compressible volume, however, has a lower stiffness than a rigid body, so it can have a smaller mass. Its resonance period is not governed by its size.

Based on this idea, a number of air-filled compressible devices have been investigated in this paper, where the variability of volume has been achieved by means of an idealized horizontal surface free to move vertically relative to the body. In total, six different device configurations have been analysed (table 1).

**Table 1.** Qualitative comparison of the six wave energy devices with compressible volumes.

device	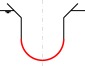	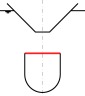	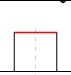	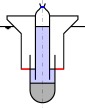	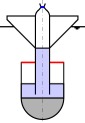	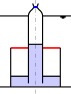
dominant radiator	body heave	body heave	moving surface	body heave	body heave	moving surface
air volume requirement	very large	small	large	small	small	large
absorbed power bandwidth	narrow	narrow	broad	very narrow	narrow	broad
water depth limited?	no	no	yes	no	no	yes

In summary, we have two design options depending on which mode should be the dominant wave radiator. If the body heave is the dominant wave radiator, the body should be in resonance, while the moving surface should not. If the moving surface is the dominant wave radiator, the moving surface should be in resonance, while the body should not. Our results suggest that the two modes should not be both in resonance. For the first option, the air volume should be small. For the second option, the air volume has to be sufficiently large. Some of this air volume can be above water.

The floating devices with downward-facing moving surfaces can only work with the first option, as the downward-facing moving surface is relatively stiff. Our findings suggest that the performance of these devices is poor. Without a water column, the air volume requirement is very large. While the addition of a water column relaxes the air volume requirement, the absorbed power bandwidth becomes narrower. For the floating devices with upward-facing moving surfaces, we have in this paper considered the first option, but it is possible to design it to work with the second option, as discussed in §6*d*. With the first option, the required air volume is small, but its bandwidth is relatively narrow. Indeed, when the body heave is the dominant wave radiator, the compressible volume tends to reduce the total wave radiated by the device, regardless of the orientation of the moving surface. For all the floating devices, however, the advantage of the compressible volume in lowering the resonance frequency of the device is clearly demonstrated: resonance happens at a frequency lower than the resonance frequency of a rigid device with equal dimensions.

A remarkably broad bandwidth is obtained for the bottom-fixed devices with upward-facing moving surface. Here, the moving surface is the only wave radiator. Devices with displaced volumes less than 2000 m^3^ and with constant turbine coefficients have been shown to be capable of achieving 80% of the theoretical maximum absorbed power over a wave period range of about 4 s. The required air volume in this case was about 2500 m^3^. In comparison, a heaving rigid semi-submerged sphere with a displaced volume of 7000 m^3^ and with a constant PTO damping attains 80% of the theoretical maximum absorbed power only over a wave period range of 2 s.

In practice, the moving surface may possibly be a rigid surface mounted on flexible bellows, or a rigid surface connected to the walls of V1 in the manner of a loudspeaker diaphragm, or it may be made completely out of a flexible membrane, as suggested in [9], §2.3.
